# Supplementary material for: A high-quality genome assembly from a single, field-collected spotted lanternfly (Lycorma delicatula) using the PacBio Sequel II system
Source: Gigascience. 2019 Oct 14;8(10):giz122. doi: 10.1093/gigascience/giz122 (PMC6791401; doi:10.1093/gigascience/giz122)
Supplement: giz122_Supplemental_Table_and_Figures [file giz122_supplemental_table_and_figures.pdf]

Materials & Methods

Sample collection and DNA isolation

A cohort of *L. delicatula* females were collected off the trunk of their preferred host *Ailanthus altissima* (tree of heaven) in Reading, Berks County, Pennsylvania, USA (40.33648 N, 75.90471 W) on the 26th of August 2018. Individuals were snap frozen in liquid nitrogen in the field and stored at -80 °C until processing. *L. delicatula* were extracted individually, by first cutting off the abdomen, and grinding the head and thorax in liquid nitrogen to a powder. High molecular weight DNA was extracted using a modification of a "salt-out" protocol described (https://support.10xgenomics.com/de-novo-assembly/sample-prep/doc/demonstrated-protocol-dna-extraction-from-single-insects). Briefly, the ground material was resuspended in 1.8 ml of lysis buffer (10 mM Tris-HCl, 400 mM NaCl, and 100 mM EDTA, pH 8.0) and 120 µl of 10% SDS and 300 ul of Proteinase K solution (1 mg/ml Proteinase K, 1% SDS, and 4 mM EDTA, pH 8.0) was added. The sample was incubated overnight at 37 °C. To remove RNA, 40 µl of 20 mg/ml RNAse A was added and the solution was incubated at room temperature for 15

minutes. Seven hundred and twenty µl of 5 M NaCl was added and mixed gently through inversion. The sample was centrifuged at 4 °C at 1500 x g for 20 minutes. A wide-bore pipette tip was then used to transfer the supernatant, avoiding any precipitated protein material, to a new tube and DNA was precipitated through addition of 3.6 ml of 100% EtOH. The DNA was pelleted at 4 °C at 6250 x g for 15 min, and all EtOH was decanted from the tube. The DNA pellet was allowed to dry and then was resuspended in 150 µl of TE. Initial quality and quantity of DNA was determined using a Qubit fluorometer and evaluating DNA on a 1% agarose genome on a Pippin Pulse using a 14-hour 5kb - 80kb separation protocol. DNA was sent to Pacific Biosciences (Menlo Park, California) for library preparation and sequencing.

Library preparation and sequencing

Genomic DNA quality was evaluated using the FEMTO Pulse automated pulsed-field capillary electrophoresis instrument (Agilent Technologies, Wilmington, DE), showed a DNA smear, with majority >20kb (Figure 2), appropriate for SMRTbell library construction without shearing.

One SMRTbell library was constructed using the SMRTbell Express Template Prep kit 2.0 (Pacific Biosciences, Menlo Park, CA). Briefly, 5 µg of the genomic DNA was carried into the first enzymatic reaction to remove single-stranded overhangs followed by treatment with repair enzymes to repair any damages that may be present on the DNA backbone. After DNA damage repair, ends of the double-stranded fragments were polished and subsequently tailed with an A-overhang. Ligation with T-overhang SMRTbell adapters was performed at 20 °C for 60 minutes. Following ligation, the SMRTbell library was purified with 1X AMPure PB beads. The size distribution and concentration of the library were assessed using the FEMTO Pulse and dsDNA BR reagents Assay kit (Thermo Fisher Scientific, Waltham, MA). Following library characterization, 3 µg was subjected to a size-selection step using the BluePippin system (Sage Science, Beverly, MA) to remove SMRTbells ≤ 15 kb. After size selection, the library was purified with 1X AMPure PB beads. Library size and quantity were assessed using the FEMTO Pulse (Figure 2), and the Qubit Fluorometer and Qubit dsDNA HS reagents Assay kit.

Sequencing primer v2 and Sequel II DNA Polymerase were annealed and bound, respectively, to the final SMRTbell library. The library was loaded at an on-plate concentration of 30 pM using diffusion loading. SMRT sequencing was performed using a single 8M SMRT Cell on the Sequel II System with Sequel II Sequencing Kit, 1800-minute movies, and Software v6.1.

Assembly

Data were assembled with FALCON-Unzip [21] using pb-falcon version 0.2.6 from the bioconda pb-assembly metapackage version 0.0.4 with the following configuration:

genome_size = 2500000000; seed_coverage = 30; length_cutoff = -1; length_cutoff_pr = 10000; pa_daligner_option = -e0.8 -l1000 -k18 -h70 -w8 -s100; ovlp_daligner_option = -k24 -h1024 -e.92 -l1000 -s100; pa_HPCdaligner_option = -v -B128 -M24; ovlp_HPCdaligner_option = -v -

B128 -M24; pa_HPCTANmask_option = -k18 -h480 -w8 -e.8 -s100; pa_HPCREPmask_option = -k18 -h480 -w8 -e.8 -s100; pa_DBsplit_option = -x500 -s400; ovlp_DBsplit_option = -s400; falcon_sense_option = --output-multi --min-idt 0.70 --min-cov 3 --max-n-read 100 --n-core 4; overlap_filtering_setting = --max-diff 100 --max-cov 200 --min-cov 3 --n-core 24; polish_include_zmw_all_subreads = true

The assembly was polished once as part of the FALCON-Unzip workflow and a second time by mapping all subreads to the concatenated reference with pbmm2 v1.1.0 (“pbmm2 align $REF $BAM $MOVIE.aln.bam --sort -j 48 -J 48”) and consensus calling with Arrow with gcpp v 0.0.1-e2ea76a (“gcpp -j 4 -r $REF -o $OUT.$CONTIG.fasta $BAM -w "$W"”). Both tools are available through bioconda: https://github.com/PacificBiosciences/pbbioconda. We screened the primary assembly for duplicate haplotypes using Purge Haplotigs (bioconda v1.0.4) [53]. Purge Haplotigs identifies candidate haplotigs in the primary contigs using PacBio read coverage depth and contig alignments. To determine the coverage thresholds, we mapped only the unique subreads to the primary contigs rather that all subreads. This resulted in more distinct modes in the coverage histogram (data not shown). A fasta file of unique subreads was generated with the command, “python -m falcon_kit.mains.fasta_filter median movie.subreads.fasta > movie.median.fasta” which is available in the pb-assembly software. We used coverage thresholds of 5, 25, and 10 and default parameters except “-s 90” (diploid coverage maximum for auto-assignment of contigs as suspect haplotigs). We recategorized 1,269 primary contigs as haplotigs (total length 141.8 Mb), discarded 12 as artifactual (total length 869 kb) and 201 as repeats (total length 19.1 Mb). A perl script (https://github.com/skingan/adapt_PurgeHaplotigs_for_FALCONPhase) was used to rename the haplotigs using the FALCON-Unzip nomenclature so that each haplotig can be easily associated with a primary contig. Following renaming, we aligned each haplotig to its associate primary contig, chained sub-alignments in one dimension, and removed redundant haplotigs whose alignment to the primary was completely contained within another haplotig [39]. This process removed 518 haplotigs totaling 22.6 Mb.

Contaminant and symbiont screening

All primary contigs from the draft FALCON assembly were searched using DIAMOND BLASTx against the NCBI nr database (downloaded April 8th, 2019) [54], and the subsequent hits were used to assign taxonomic origin of each contig using a least common ancestor assignment for each contig utilizing MEGAN 6.15.2 Community Edition with the longReads LCA Algorithm and readCount assignment mode [55]. Any contigs that were identified as microbial were flagged and removed from the final assembly. To avoid assignment of contigs as microbial when a microbial gene may have horizontally transferred to the insect, any potentially microbial contigs were screened for presence of BUSCO insect genes and retained if a BUSCO was present on the contig.

Genome assembly evaluation

To assess the completeness of the curated assembly, we searched for conserved, single copy genes using BUSCO (Benchmarking Universal Single-Copy Orthologs, BUSCO, RRID:SCR_015008) v3.0.2 [27] with the 'insecta_odb9' database. In addition, we evaluated assembly completeness and accuracy against the *Drosophila melanogaster* CEGMA gene set (http://korflab.ucdavis.edu/datasets/cegma/core_genome/D.melanogaster.aa), using a previously described script [56]. A visualization of the assembly contiguity and completeness was generated using assembly-stats [26] and are presented in Figure 3 and Table 1.

We also applied an orthogonal method to estimate the genome size by dividing the total base pairs of unique subreads (82.4 Gb) by the modal read coverage (30-fold, Figure S2) of the PacBio data. This calculation is possible because PacBio data has minimal sequencing bias across DNA content and sequence complexity [57, 58]. Unique subreads were mapped to the curated primary assembly ("minimap2 -ax map-pb $REF $QRY --secondary=no" [59], read depth was estimated with "bedtools genomecov" [60], and a histogram was visualized in R [61].

Availability of Data

Raw data and final assembly for this project are submitted to NCBI under BioProject PRJNA540533, sample described in BioSampleSAMN11546444, SRA accession for raw PacBio subreads (fastq formatted) is SRR9005207. Supporting data to this publication is submitted to the AgDataCommons, including polished FALCON assembly, polished FALCON-Unzip assembly, final curated assembly and placement file, microbial symbiont assemblies and associated metadata[62]. Additional supporting data and materials are available in the *GigaScience* GigaDB database [63].

Additional Files

Gene Count	FALCON-Unzip Primary (haplotigs)	Purge Haplotigs Primary (haplotigs)	Final Curated Primary (haplotigs)
Complete	1602 (954)	1604 (977)	1604 (975)
Duplicate	54 (12)	40 (16)	40 (14)
Single Copy	1548 (942)	1564 (961)	1564 (961)
Fragmented	33 (110)	30 (99)	30 (101)
Missing	23 (594)	24 (582)	21 (582)

Table S1: Full summary from BUSCO analysis of primary contigs, using the 'insecta_odb9' gene set (Total = 1658), after different stages of assembly and curation.

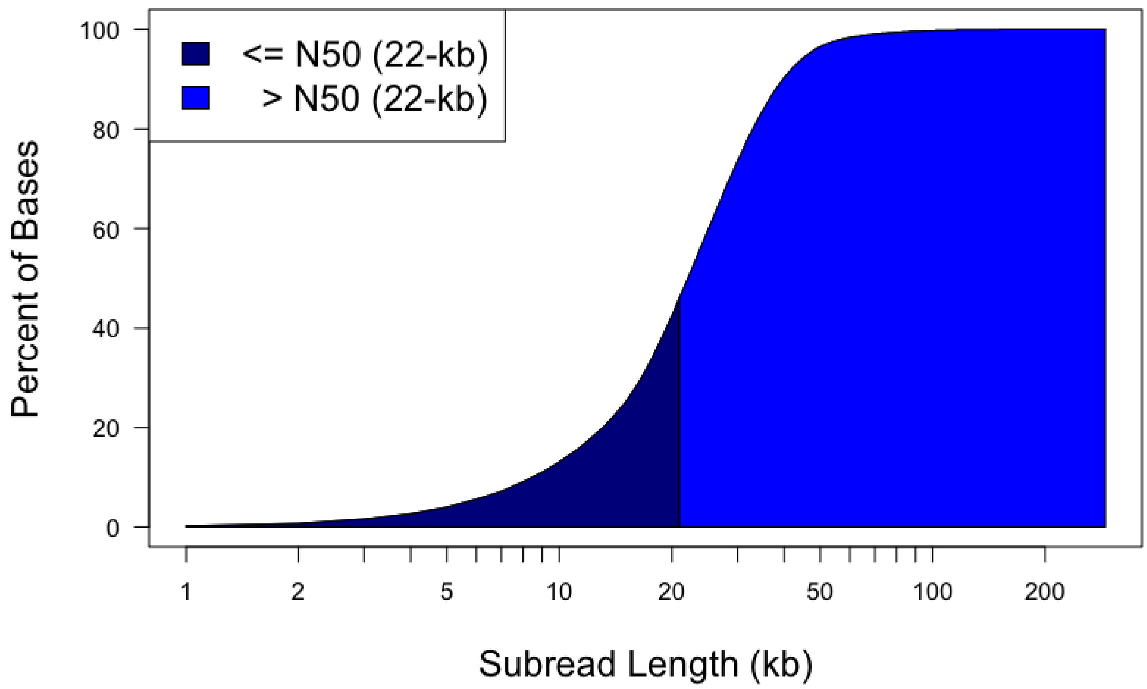

Figure S1: **Cumulative distribution of subread lengths for Sequel II 8M SMRT Cell of 15-kb size-selected library.** Data were bioinformatically filtered prior to assembly to remove reads shorter than 500-bp and retain one subread per library molecule (see methods).

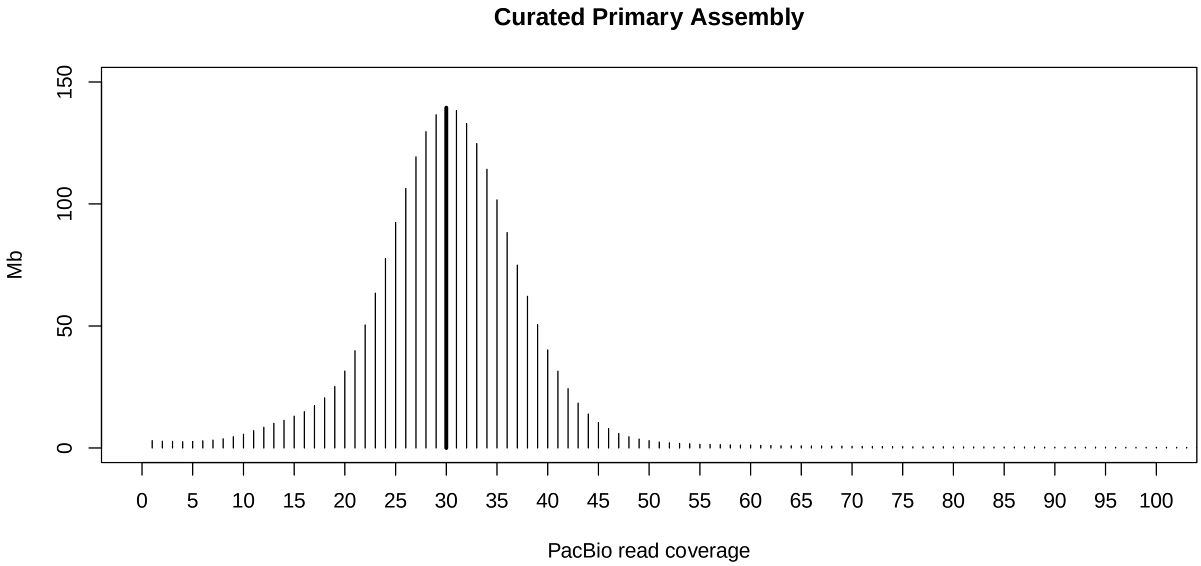

Figure S2: **Coverage depth histogram.** PacBio reads mapped to curated primary contigs shows unimodal coverage with peak centered at 30-fold.
