# Supplementary material for: A high-quality genome assembly from a single, field-collected spotted lanternfly (Lycorma delicatula) using the PacBio Sequel II system
Source: Gigascience. 2019 Oct 14;8(10):giz122. doi: 10.1093/gigascience/giz122 (PMC6791401; doi:10.1093/gigascience/giz122)
Supplement: giz122_Response_to_Reviewer_Comments_Original_Submission [file giz122_response_to_reviewer_comments_original_submission.pdf]

Author's Response To Reviewer Comments

Close

Detailed responses to the reviewers' comments:

We would like to thank reviewer #1 for the enthusiastic response and positive feedback about the content and scope of our manuscript.

We would like to thank reviewer #2 for the comments and suggestions, which we have addressed below, and we have applied changes to the manuscript also outlined below to accommodate the reviewer's suggestions. We have added an orthogonal method of estimating the genome size, leveraging the minimal bias of PacBio sequencing data and thereby allowing the genome size estimate from the read coverage. We have added language pertaining to this addition in the Results section, the Materials & Methods section, and with an additional supplementary Figure (Fig. S2), respectively, confirming that the assembly is not overly redundant and consistent with the assembly genome size estimate:

"and an orthogonal method to estimate the genome size using read coverage depth."
"Using read coverage (see Figure S2 and Material and Methods), we also generated a genome size estimate of 2.75 Gb which is slightly larger than our curated primary assembly, consistent with telomeric, centromeric and rDNA satellite regions being refractory to genome assembly (https://www.ncbi.nlm.nih.gov/pmc/articles/PMC6274785/, https://genomebiology.biomedcentral.com/articles/10.1186/gb-2001-2-7-research0025, https://www.genetics.org/content/genetics/211/1/333.full.pdf)."

"We also applied an orthogonal method to estimate the genome size by dividing the total base pairs of unique subreads (82.4 Gb) by the modal read coverage (30-fold, Figure S2) of the PacBio data. This calculation is possible because PacBio data has minimal sequencing bias across DNA content and sequence complexity (https://genomebiology.biomedcentral.com/articles/10.1186/gb-2013-14-5-r51, https://www.nature.com/articles/nrg3933). Unique subreads were mapped to the curated primary assembly ("minimap2 -ax map-pb $REF $QRY --secondary=no", https://academic.oup.com/bioinformatics/article/34/18/3094/4994778), read depth was estimated with "bedtools genomecov" (https://currentprotocols.onlinelibrary.wiley.com/doi/abs/10.1002/0471250953.bi1112s47), and a histogram was visualized in R (R Development Core Team (2008). R: A language and environment for statistical computing. R Foundation for Statistical Computing, Vienna, Austria. ISBN 3-900051-07-0, URL http://www.R-project.org.).

Figure S2: Coverage depth histogram. PacBio reads mapped to curated primary contigs shows unimodal coverage with peak at 30-fold.

The reviewer's comment about "paralogous genes, such as gene expansion and contraction" addresses a different aspect of genome biology requiring functional gene annotation, which is outside the scope of our manuscript and aren't really related to assembly quality. It is distinct from our demonstration of a diploid representation of the genome, with haplotypes well separated, and thereby limiting the common redundancy/inflation of genome size by using the latest advances in long-read genome assembly.

Reviewer #2 suggested 'toning down' some of the technological and methodological advances highlighted in the paper, however, due to the request by the editor to "emphasize the advantages of data from this new PacBio sequencer on entomological genomics", we have left these aspects unchanged. One of the primary points of the paper is the ability to obtain significantly more data from the same starting tissue of insect (and same DNA amount), thus opening the door to sequencing single insects, where previously one would have needed to pool multiple individuals. We believe that we have not made any claims as to the generality or expandability of the described procedures beyond what is described in the manuscript.

With regard to the note about a “high-quality genome assembly with cutting-edge techniques”, we would like to respectfully contend that a haplotype-resolved, diploid falcon-unzip assembly represents a cutting-edge high-quality genome assembly, particularly in insect genomics, surpassing previous efforts, illustrated in Table 2, in a number of important genome quality and workflow aspects. Our intent was to rapidly publish a high-quality genome of a rapidly emerging agricultural invasive species as a resource for other researchers involved in combating this threat. In addition, the submitted genome will aid researchers studying the basic biology of hemipterans which has been an underrepresented clade with regard to high-quality genomic resources. From the feedback by reviewer #2, we realize that the submission categorization of “Research” was not a good fit for this intent, and we therefore propose to redesignate the submission category as “Data Note”, which according to the description on your journal website (https://academic.oup.com/gigascience/pages/data_note) appears to be much better suited to the scope of our work for several reasons. First, the website description for Data Note includes: “One of the aims of a Data Note is to incentivize and more rapidly release data before subsequent detailed analysis has been carried out,” which is one of the intended goals of our study while work towards a final, scaffolded and annotated reference genome is ongoing. In addition, the Data Note category highlights a focus on “Novel technology or methodology used to create dataset.” As summarized in the Discussion section of our manuscript, the work describes such novel technology and methodology in four separate areas critical to entomological genome research, namely:

Collection strategies: the possibility of obtaining high-quality genomes from single, wild-caught individuals, thereby obviating the need of laborious and time-consuming lab strain cultures
Library preparation efforts and sequencing time: to our knowledge, this is the first paper utilizing the new Sequel II sequencing system (although there are by now several other examples of this in the bioarchives so the priority will depend on the date of official publication between this manuscript and these others)
Assembly: the described work highlights a powerful example of the paradigm shift towards highly heterozygous, outbred individuals as the most optimal specimen for genome projects, overturning the long-held belief that inbreeding is optimal
Endosymbiont genome capture: previous work in this area has relied on laborious and time-consuming isolation of the endosymbiont tissue and separate preparation, sequencing and assembly. Our work demonstrates that this important information can be obtained simultaneously with the host organism.

Third, one of the featured aspects of the “Data Note” format in Gigascience is stated as a “Need for immediate public health issues.” While the invasion of the spotted lanternfly does not represent a direct threat to human health, it is clearly a dramatic and imminent threat to the agricultural industry and tourism in the Eastern United States and beyond. This was, among other outlets, powerfully highlighted by a feature report by the Pennsylvania USDA branch, available at https://www.youtube.com/watch?v=qLMCSBjpOIc. The video describes that the spotted lanternfly is the worst invasive species the U.S. has seen in 150 years, and threatening industries that tally to an estimated ~18 billion dollars. The acquisition of additional data, including Illumina data, RNA, Bionano, Hi-C and potential others as suggested by reviewer #2 will take time (currently, this species cannot be artificially reared, so RNAseq data collection relies on the wild development of this insect in order to procure a robust sampling across developmental stages to support annotation), including the subsequent analysis thereof and curation of the final reference genome. But the accessibility of this assembly will directly impact current and emerging research across many research groups who are tackling the response of this invasive pest. We therefore propose to change the categorization of the manuscript to a Data Note which fits much better with the website description and the expectation of reviewer #2 regarding the scope of our work presented.

Close
