# Supplementary material for: A high-quality genome assembly from a single, field-collected spotted lanternfly (Lycorma delicatula) using the PacBio Sequel II system
Source: Gigascience. 2019 Oct 14;8(10):giz122. doi: 10.1093/gigascience/giz122 (PMC6791401; doi:10.1093/gigascience/giz122)
Supplement: giz122_Response_to_Reviewer_Comments_Revision_1 [file giz122_response_to_reviewer_comments_revision_1.pdf]

Author's Response To Reviewer Comments

Close

Response to review for GIGA-D-19-00171R1
"A High-Quality Genome Assembly from a Single, Field-collected Spotted Lanternfly (Lycorma delicatula) using the PacBio Sequel II System"
Comments from Scott Edmunds (Editor)

To summaries our thoughts on the changes, if you have any remaining DNA and RNA from the single individual, then it would make sense to improve the genome with transcriptome or short read sequencing. If you don't, then more discussion is required to both sell the advantages of this new platform, and better explain the limitations regarding the increased errors (InDels, etc., and the implications of these) resulting from this data. As you will have seen in Mark Blaxter's talk at the Genomes2019 meeting this week there is an important need for technologies to "sequence the tinies", and this single Sequel II flowcell approach potentially fills that niche. A bit more work is definitely needed to explain that in the text, so please make sure that is properly stressed in the introduction and conclusions. As the lanternfly continues to spread in the US (see https://www.pennlive.com/life/2019/08/spotted-lanternflys-big-move-is-here.html), and up-to-date reports of this would also be good to help set the scene and stress the urgency.

Response: While we don't plan to include RNAseq data in this manuscript, we have expanded the discussion of the advantages of the PacBio Sequel II system and its applications to sequence the "tinies". We've also expanded the discussion of the Spotted lanternfly as an expanding pest in the Northeastern U.S.

Comments from Shanlin Liu (Reviewer 2)
The authors want to keep their statements of those advantages on entomological genomics. Although I still consider all these advantages are derived from the upgrade of Pacbio SMRT sequencing system, I have no objection if the authors insist on it. This is not the issue I am most worried about. The authors obtained this genome using only long reads, as I mentioned in my comments the last round, this is an incomplete genome assembly. The authors also think this paper should be categorized as Data Note, however, it is well known that genome assembled using only the error-prone long reads tends to have lots of small-scale errors like InDels which will introduce frameshift and premature stop codons and affect the interpretation of translated regions. The authors can find more details in a paper recently published on NBT (https://doi.org/10.1038/s41587-018-0004-z), it mentioned that the human genome assembly generated using only the Pacbio long reads included the most errors compared to the other assembly, with thousands of protein-coding genes predicted to be disrupted by indels. Therefore, the authors may want to include shortgun reads to polish this genome and correct those potential and critical errors before its publication. In addition, you may want to include some transcriptome data as well to improve the genome annotation if you such dataset.

Response: While we did not generate RNAseq data in this data note, we did assess for accuracy of the assembly by mapping core genes to the assembly and assessing for frame shift errors.

"As an additional evaluation, we aligned to the primary assembly the core Drosophila melanogaster CEGMA gene set, resulting in 416 alignments (91%) and an average alignment length of 86%, and with >96.6% of alignments showing no frame shift-inducing indels."
This suggests that while the assembly is not without any errors, it is of sufficient quality to use as a foundation for genomics in this species. Some components of our approach that allows for high consensus accuracy from single molecule data is through updated sequencing chemistry (P6-C4), as well as utilization of multiple rounds of the arrow polishing tool, and the ability of this tool to utilize multiple subreads from a single sequencing reaction together to reach a higher consensus accuracy than previously allowed (i.e. CCS polishing, an option added in Falcon_unzip 1.1.5 in the Spring of 2019 using option "polish_include_zmw_all_subreads = true"). Consensus accuracy appears to be on par with similar assemblies found in Gigascience and other published genomes using long read technology and of sequencing goals set by the Earth Biogenome Project. In future versions of this genome, we anticipate

further assembly/scaffolding and gene model construction with RNAseq data.

Close
