# Supplementary material for: A high-quality genome assembly from a single, field-collected spotted lanternfly (Lycorma delicatula) using the PacBio Sequel II system
Source: Gigascience. 2019 Oct 14;8(10):giz122. doi: 10.1093/gigascience/giz122 (PMC6791401; doi:10.1093/gigascience/giz122)
Supplement: giz122_Reviewer_2_Report_Original_Submission — Shanlin Liu -- 7/9/2019 Reviewed [file giz122_reviewer_2_report_original_submission.pdf]

Reviewer Report

Title: A High-Quality Genome Assembly from a Single, Field-collected Spotted Lanternfly (Lycorma delicatula) using the PacBio Sequel II System

Version: Original Submission **Date:** 7/9/2019

Reviewer name: Shanlin Liu

Reviewer Comments to Author:

The authors present us a genome assembly for Spotted Lanternfly using the new sequencing platform Pacbio Sequel II. The genome quality is okay but requires further improvements.
Firstly, the authors may want to estimate its genome size using either a K-mer based analysis or flow cytometry. Although the BUSCO results showed good coverage of the coding regions, we can hardly conclude whether the assembly is redundant or not, especially for genome analysis regarding paralogous genes, such as gene expansion and contraction.
Then, those improvements claimed by the authors in the current study, such as sample collection, single individual based DNA extraction and sequencing, stem from the upgrade of the sequencer, "Pacbio Sequel II" which can generate eight million long reads per flow cell, rather than the half a million long reads of its previous version. Plus, the biomass of a single individual will considerably influence the DNA yield and then alter those improvements proposed by the authors. You may want to tone down them in your manuscript.
Additionally, as a data note publication without scientific hypothesis being resolved, the authors may want to release a high-quality genome assembly with cutting-edge techniques, such as Bionano, Hi-C. And RNA-Seq should also be included to improve the annotation quality.

Level of Interest

Please indicate how interesting you found the manuscript: Choose an item.

Quality of Written English

Please indicate the quality of language in the manuscript: Choose an item.

Declaration of Competing Interests

Please complete a declaration of competing interests, considering the following questions:

- Have you in the past five years received reimbursements, fees, funding, or salary from an organisation that may in any way gain or lose financially from the publication of this manuscript, either now or in the future?

- Do you hold any stocks or shares in an organisation that may in any way gain or lose financially from the publication of this manuscript, either now or in the future?
- Do you hold or are you currently applying for any patents relating to the content of the manuscript?
- Have you received reimbursements, fees, funding, or salary from an organization that holds or has applied for patents relating to the content of the manuscript?
- Do you have any other financial competing interests?
- Do you have any non-financial competing interests in relation to this paper?

If you can answer no to all of the above, write 'I declare that I have no competing interests' below. If your reply is yes to any, please give details below.

I declare that I have no competing interests

I agree to the open peer review policy of the journal. I understand that my name will be included on my report to the authors and, if the manuscript is accepted for publication, my named report including any attachments I upload will be posted on the website along with the authors' responses. I agree for my report to be made available under an Open Access Creative Commons CC-BY license (http://creativecommons.org/licenses/by/4.0/). I understand that any comments which I do not wish to be included in my named report can be included as confidential comments to the editors, which will not be published.

Choose an item.

To further support our reviewers, we have joined with Publons, where you can gain additional credit to further highlight your hard work (see: https://publons.com/journal/530/gigascience). On publication of this paper, your review will be automatically added to Publons, you can then choose whether or not to claim your Publons credit. I understand this statement.

Yes Choose an item.
