# Supplementary material for: A high-quality genome assembly from a single, field-collected spotted lanternfly (Lycorma delicatula) using the PacBio Sequel II system
Source: Gigascience. 2019 Oct 14;8(10):giz122. doi: 10.1093/gigascience/giz122 (PMC6791401; doi:10.1093/gigascience/giz122)
Supplement: giz122_Reviewer_2_Report_Revision_1 — Shanlin Liu -- 8/28/2019 Reviewed [file giz122_reviewer_2_report_revision_1.pdf]

Reviewer Report

Title: A High-Quality Genome Assembly from a Single, Field-collected Spotted Lanternfly (Lycorma delicatula) using the PacBio Sequel II System

Version: Revision 1 **Date:** 8/28/2019

Reviewer name: Shanlin Liu

Reviewer Comments to Author:

The authors want to keep their statements of those advantages on entomological genomics. Although I still consider all these advantages are derived from the upgrade of Pacbio SMRT sequencing system, I have no objection if the authors insist on it. This is not the issue I am most worried about. The authors obtained this genome using only long reads, as I mentioned in my comments the last round, this is an incomplete genome assembly. The authors also think this paper should be categorized as Data Note, however, it is well known that genome assembled using only the error-prone long reads tends to have lots of small-scale errors like InDels which will introduce frameshift and premature stop codons and affect the interpretation of translated regions. The authors can find more details in a paper recently published on NBT (https://doi.org/10.1038/s41587-018-0004-z), it mentioned that the human genome assembly generated using only the Pacbio long reads included the most errors compared to the other assembly, with thousands of protein-coding genes predicted to be disrupted by indels. Therefore, the authors may want to include shortgun reads to polish this genome and correct those potential and critical errors before its publication. In addition, you may want to include some transcriptome data as well to improve the genome annotation if you such dataset.

Level of Interest

Please indicate how interesting you found the manuscript: Choose an item.

Quality of Written English

Please indicate the quality of language in the manuscript: Choose an item.

Declaration of Competing Interests

Please complete a declaration of competing interests, considering the following questions:

- Have you in the past five years received reimbursements, fees, funding, or salary from an organisation that may in any way gain or lose financially from the publication of this manuscript, either now or in the future?
- Do you hold any stocks or shares in an organisation that may in any way gain or lose financially from the publication of this manuscript, either now or in the future?

- Do you hold or are you currently applying for any patents relating to the content of the manuscript?
- Have you received reimbursements, fees, funding, or salary from an organization that holds or has applied for patents relating to the content of the manuscript?
- Do you have any other financial competing interests?
- Do you have any non-financial competing interests in relation to this paper?

If you can answer no to all of the above, write 'I declare that I have no competing interests' below. If your reply is yes to any, please give details below.

I declare that I have no competing interests

I agree to the open peer review policy of the journal. I understand that my name will be included on my report to the authors and, if the manuscript is accepted for publication, my named report including any attachments I upload will be posted on the website along with the authors' responses. I agree for my report to be made available under an Open Access Creative Commons CC-BY license (http://creativecommons.org/licenses/by/4.0/). I understand that any comments which I do not wish to be included in my named report can be included as confidential comments to the editors, which will not be published.

Choose an item.

To further support our reviewers, we have joined with Publons, where you can gain additional credit to further highlight your hard work (see: https://publons.com/journal/530/gigascience). On publication of this paper, your review will be automatically added to Publons, you can then choose whether or not to claim your Publons credit. I understand this statement.

Yes Choose an item.
